# Tobacco and marijuana use and their association with serum prostate-specific antigen levels among African American men in Chicago

**DOI:** 10.1016/j.pmedr.2020.101174

**Published:** 2020-08-11

**Authors:** David J. Press, Brandon Pierce, Diane S. Lauderdale, Briseis Aschebrook-Kilfoy, Scarlett Lin Gomez, Donald Hedeker, Nathaniel E. Wright, Richard J. Fantus, Luís Bettencourt, Habibul Ahsan, Scott Eggener

**Affiliations:** a Department of Public Health Sciences, University of Chicago, Chicago, IL, USA; b Department of Preventive Medicine, Northwestern University Feinberg School of Medicine, Chicago, IL, USA; c The Center for Health Information Partnerships (CHiP), Institute of Public Health & Medicine, Northwestern University Feinberg School of Medicine, Chicago, IL, USA; d Department of Human Genetics, University of Chicago, Chicago, IL, USA; e Institute for Population and Precision Health, University of Chicago, Chicago, IL, USA; f Department of Epidemiology and Biostatistics, University of California, San Francisco, School of Medicine, San Francisco, CA, USA; g Medical Scientist Training Program, Pritzker School of Medicine, University of Chicago, Chicago, IL, USA; h Department of Surgery, University of Chicago, Chicago, IL, USA; i Mansueto Institute for Urban Innovation, University of Chicago, Chicago, IL, USA; j Department of Ecology and Evolution, University of Chicago, Chicago, IL, USA; k Department of Sociology, University of Chicago, Chicago, IL, USA; l Santa Fe Institute, Santa Fe, NM, USA; m Department of Medicine, University of Chicago, Chicago, IL, USA

**Keywords:** Prostate specific antigen, Cigarette, Tobacco, Marijuana, African American

## Abstract

African American (AA) men experience more than twice the prostate cancer mortality as White men yet are under-represented in academic research involving prostate-specific antigen (PSA), a biomarker of prostate cancer aggressiveness. We examined the impact of self-reported tobacco (cigarette pack-years and current tobacco use including e-cigarettes) and current regular marijuana use on serum PSA level based on clinical laboratory testing among 928 AA men interviewed 2013–2018 in Chicago. We defined outcome of elevated PSA ≥ 4.0 ng/mL for logistic regression models and continuous PSA increases for general linear models. All models were adjusted for age, sociodemographic characteristics, healthcare utilization, body mass index, and self-reported health. Among 431 AA men age ≥ 55 years, we observed ∼ 5 times the odds of elevated PSA among those with > 1 pack-years of cigarette smoking vs. never-smokers (odds ratio [OR] = 5.09; 95% confidence interval [CI] = 1.57–16.6) and a quarter the odds of elevated PSA among current marijuana users vs. non-users (OR = 0.27; 95% CI = 0.08–0.96). PSA increased on average 1.20 ng/mL among other current tobacco users vs. non-users. Among older AA men, cigarette smoking history and current tobacco use were positively associated with an increase in PSA levels and current marijuana use were inversely associated with PSA levels. Future work with studies of diverse patient populations with cancer outcomes are needed to assess whether these behavioral characteristics contribute to racial/ ethnic disparities in prostate cancer outcomes. Our study provides novel evidence regarding potential differences in PSA levels among older AA men according to behavioral characteristics.

## Highlights

• AA men are under-represented in PSA research, a biomarker of prostate cancer aggresiveness.
• Cigarette smoking was associated with an increase in PSA among older AA men.
• Tobacco use was associated with an increase in PSA among older AA men.
• Marijuana use was associated with a decrease in PSA among older AA men.
• Future behavioral risk factor studies linked to biopsy outcomes are warranted.

## 1 Introduction

Prostate-specific antigen (PSA) is a glycoprotein molecule involved in liquefaction of seminal fluid (Wang et al., 1979). Sociodemographic and anthropometric factors associated with increasing PSA levels in males include advanced age, African American (AA) race/ ethnicity, low body mass index (BMI), and greater height (Bonn et al., 2016). Known clinical correlates of serum PSA include non-malignant characteristics such as larger prostate volume, inflammation, infection, trauma, and medical procedures involving the prostate gland (Nadler et al., 1995, Malati et al., 2006, Ulleryd et al., 1999, Kravchick et al., 2007, Lechevallier et al., 1999, Moyer, 2012). Importantly, increased PSA is a strong predictor of aggressive prostate cancer (Carter et al., 1992, Catalona et al., 2000, Schröder et al., 2012, MacKintosh et al., 2016;6:157-, Loeb et al., 2012)and about 90% of prostate cancer deaths occur among men with PSA > 2 ng/ mL at age 60 (Carlsson et al., 2014, Cuzick et al., 2014). However, there is limited research on PSA test performance in AA populations, who experience distinct age-specific prostate cancer risk distributions (Wolf et al., 2010, Verges et al., 2017, US Preventive Services Task Force, 2018).

Relative to White men, AA men with prostate cancer present with higher PSA at presentation, greater overall tumor volumes per ng/ mL of serum PSA, and more aggressive disease (Moul et al., 1999, Moul et al., 1995, Sutcliffe et al., 2012, Sanchez-Ortiz et al., 2006)and experience more than twice the prostate cancer mortality (Siegel et al., 2018). Racial/ ethnic disparities in prostate cancer aggressiveness and mortality within the AA population are likely due to complex biological, socioeconomic, and socio-cultural determinants underlying disparities in presentation, diagnosis, treatment, and survival (Chornokur et al., 2011). Recent United States Preventives Services Task Force (USPSTF) acknowledged the AA population as a high risk group and recommended shared decision-making regarding PSA testing (Moyer VA on behalf of the U.S. Preventive Services Task Force, 2012, US Preventive Services Task Force Recommendation statement, 2018). Yet AA men are less likely to be informed about the option of a PSA test, less likely to report a PSA test in the past year (American Cancer Society., 2016), less likely to use primary care (Arnett et al., 2016), and are under-represented in academic research involving PSA measurements (Schröder et al., 2012, Loeb et al., 2012, Henderson et al., 1997, Connolly et al., 2008, Andriole et al., 2005). AA men are also exposed to disproportionately high levels of comorbid conditions as well as residence in low socioeconomic status (SES) neighborhoods, which are independently associated with PSA levels (Shaikh et al., 2015, Ferdinand et al., 2017, Morris et al., 2010, Firebaugh and Acciai, 2016, Jackson et al., 2010). Moreover, it remains unclear whether differences in biology as opposed to differences in general male health or risk factors among men residing in low SES neighborhoods may contribute to racial/ ethnic disparities in prostate cancer aggressiveness and mortality among AA men.

One behavioral risk factor for which there is some evidence of an association with prostate cancer risk is smoking, including tobacco and marijuana use (Kenfield et al., 2011, Huncharek et al., 2010, Ramos and Bianco, 2012, Nichols et al., 2019). These behaviors are related to age and SES. Individuals with lower SES and those residing in lower SES neighborhoods are more likely than those of higher individual SES or residing in higher SES neighborhoods to be cigarette smokers (Cambron et al., 2018, Siahpush et al., 2010), smoke cigarettes heavily (Cambron et al., 2018), are less likely to use electronic cigarettes (e-cigarettes) or premium cigars (Hartwell et al., 2017), and are more likely to use marijuana (Peters et al., 2018). However, to our knowledge, no previous studies have examined the association of these behavioral factors on PSA levels in a non-clinical study population with sizable representation from AA men. In order to examine the association between cigarette smoking, other tobacco use, and marijuana use on serum PSA levels, we conducted a cross-sectional study with clinical laboratory testing of serum PSA in the *Chicago Multiethnic Prevention And Surveillance Study* (COMPASS).

## 2 Methods

COMPASS is a population-based longitudinal cohort study with ongoing recruitment. Methods of COMPASS have been described elsewhere (Press et al., 2020). Briefly, individuals were considered eligible for inclusion in COMPASS if they were a resident of the Chicago metropolitan area, age 35 years or older, male or female, English or Spanish speaking, competent to give consent, and permanent resident or citizen of the US. Recruitment strategies to increase minority enrollment have included a predominantly minority interviewer team and focus on recruitment in census tracts with minority and diverse populations as the primary sampling unit. The present study is a cross-sectional study comprised of the first 954 AA men enrolled in COMPASS between 2013 and 2018 in Chicago. Participants were interviewed in person, generally in their homes. We conducted clinical laboratory testing of bio-specimens (including total PSA) using 0.5 mL of blood stored in gold top vacutainer tubes (SST-Serum separator) using blood samples collected from participants. Blood collection occurred at the same time as consent and the in-person interview. We excluded < 5 participants with previous prostate surgery, <5 outliers with very high PSA values above 25 ng/ mL that skewed the distribution for the purposes of statistical analyses, <5 who reported use of 5-alpha-reductase inhibitors (e.g. finasteride, dutasteride), and 15 who resided in census tracts outside of the City of Chicago. In total, we excluded 26 (2.7%) for a total sample size of 928 AA men in our analysis. Participants were not notified of their clinical laboratory test results for serum PSA. Our human subjects research study protocol was approved by the Institutional Review Board of the Biological Sciences Division at the University of Chicago.

## 3 Participant characteristics

We ascertained cigarette smoking history by self-report to 13 items, including 2 on smoking history (‘do you currently smoke cigarettes [NOT including pipes, snuff, chewing tobacco, or any other forms of tobacco besides cigarettes]?’ and ‘did you ever smoke cigarettes regularly?’) and 11 items on pack-years for ever-smokers that included average per day cigarette consumption currently and in the past. We ascertained current marijuana use by self-report to a single question (‘do you currently smoke marijuana?’). We ascertained other current tobacco use by self-report to a single question (‘do you use any of the other following tobacco products regularly now? (select all that apply)’, separately including ‘Cigar’, ‘E Cigarette’, ‘Pipe’, ‘Snuff’, ‘Chewing Tobacco’, and ‘Hookah’). Cigarillos and blunts were not directly queried but were considered by interviewers to be captured in the cigar category.

We ascertained information by self-report on age, gender, race, ethnicity, marital status, SES, healthcare utilization (including previous cancer diagnosis [yes/ no], timing of last PSA test, and timing of last digital rectal exam [DRE]), self-rated health, and hypertension medication use. We ascertained visits to a doctor in the last 12 months based on responses to three items: ‘during the past 12 months, how many times have you seen a doctor or other health care professional about your health at a… doctor’s office or clinic’, ‘…hospital emergency room’, ‘…home or some other place’. These were combined and categorized into quintiles. These healthcare utilization factors were also considered separately. Participants were asked to show the interviewer all the medications and supplements they currently use, and these were recorded. This information, together with medication questions in the interview, were used to ascertain hypertension medication use. Hypertension medication was defined as either blood pressure medication described in general terms or specific hypertension medications mentioned or presented (e.g., hydrochlorothiazide, lisinopril, which may also be used for cardio-protection after myocardial infarction or kidney stone disease). We ascertained health insurance type based on response to a single item measure. Individual SES was defined using a composite of education, employment status, and income developed by the US Department of Justice (i.e. National Crime Victimization Survey Index 3). Specifically, participants were trichotimized according to the equation (ordinal education + ordinal income + 1 if employed) (Berzofsky et al., 2014). BMI was obtained by direct height and weight measurement.

Neighborhood SES was defined by participant address. We used a publicly available measure of census tract level SES based on the 1990 and 2000 U.S. Censuses and the 2008–2012 American Community Survey, which ranges from 0 to 100 with 50 being the national average. The authors, Miles et. al., used an unconstrained single factor model according to the equation (1[ln{median household income}])+(-1.129[ln{% female-headed households}])+(-1.104[ln{%workers ≥ 16 years who are unemployed}])+(-1.974[ln{% of households in poverty}]) + 0.451([% high school grads but not bachelors holders] + 2[% bachelors holders]) (Miles et al., 2016). This continuous measure was then scaled into quintiles based on the distribution of the Chicago metropolitan area level.

## 4 Statistical analyses

We conducted analyses for two outcomes: 1) binary PSA as < 4 ng/ mL or ≥ 4 ng/ mL and 2) continuous PSA. Descriptive analyses were conducted to assess the relationship between each exposure variable, covariate, and PSA level, using chi-squared p-values for binary PSA and one-way analysis of variance (ANOVA) for continuous PSA. We examined the associations between self-reported cigarette smoking history, current use of other tobacco products, and current use of marijuana on PSA, controlling for potential confounders in multivariable logistic regression models with the outcome of binary PSA and general linear models (GLMs) with the outcome of PSA. Models were adjusted for age, marital status, individual and neighborhood SES, self-reported health, hypertension medication, BMI, health insurance type, previous cancer diagnosis, timing of last PSA test, timing of last DRE, and visits to a doctor in the last 12 months. We stratified our models by age (40–54 years and ≥ 55 years). Age 55 years was selected for stratification as some guidelines, like the American Urological Association guidelines, recommend starting PSA screening in average-risk individuals at that age (US Preventive Services Task Force, 2018, Carter et al., 2013). We further conducted sensitivity analyses examining different PSA cutoffs (i.e., 2.5, 4.0 and 10.0 ng/ mL), age cutoffs (i.e., 45, 50, and 55 years) and history of previous cancer (yes/ no). Additionally, for robustness tests, we developed a third set of analyses with natural log-transformed PSA as the outcome (continuous) using the same approach as for untransformed PSA as the outcome. We considered a nominal p < 0.05 as statistically significant for all analyses. All regression analyses were conducted using SAS version 9.4 (Cary, NC).

## 5 Results

The study sample was comprised of AA men who were 77.6% low SES, 87.2% insured by Medicaid, other government supported insurance or uninsured, and 90.1% residing in the lowest three quintiles of neighborhood SES. Mean PSA for the study sample was 1.51 ng/ mL (standard deviation [SD] = 2.28). Elevated PSA ≥ 4 ng/ mL was measured for 68 AA men (7.3%). Statistically significant differences in elevated PSA ≥ 4 ng/ mL were observed across categories of age (p < 0.001) and cigarette smoking history (p = 0.008). Statistically significant differences in mean PSA were observed across categories of age (p < 0.001) and other current tobacco use (p = 0.038)(Table 1). Participant characteristics stratified by age are provided in Supplemental Tables 1 and 2.

**Table 1:** Participant characteristics and mean prostate-specific antigen (PSA; ng/mL) level among 928 African American (AA) men, Chicago 2013–2018.

Participant Characteristics	n	Dichotomous		Continuous	
		PSA <4 (row %)	PSA ≥4 (row %)	Mean PSA	St. Dev.
Behavioral factors					
Cigarette smoking history^a^					
Never	198	95.5%	4.5%	1.40	2.04
0 to 1 pack-year	605	93.1%	6.9%	1.47	2.27
>1 pack-year	125	86.4%	13.6%	1.86	2.64
χ^2^ p-value^b^			0.008		
One-way ANOVA p-value^c^					0.168
Other current tobacco use^a^^,^^d^					
No	837	93.1%	6.9%	1.45	2.08
Yes	91	89.0%	11.0%	1.98	3.62
χ^2^ P-value^b^			0.158		
One-way ANOVA p-value^c^					0.038
Current marijuana use					
No	741	91.9%	8.1%	1.56	2.38
Yes	187	95.7%	4.3%	1.27	1.84
χ^2^ P-value^b^			0.073		
One-way ANOVA p-value^c^					0.119
Co-variates					
Age (years)^a^					
40 to <45	108	98.2%	1.8%	0.99	0.94
45 to <50	176	97.2%	2.8%	0.97	1.06
50 to <55	213	93.4%	6.6%	1.42	2.31
55 to <60	196	90.3%	9.7%	1.69	2.44
≥60	235	88.1%	11.9%	2.07	2.98
χ^2^ p-value^b^			<0.001		
One-way ANOVA p-value^c^					<0.001
Marital status^a^					
Single, never married	450	94.7%	5.3%	1.34	2.07
Married	158	92.4%	7.6%	1.57	2.21
Living with partner	53	90.6%	9.4%	1.70	1.77
Separated	74	91.9%	8.1%	1.50	2.56
Divorced	156	87.8%	12.2%	1.93	2.99
Widowed	34	94.1%	5.9%	1.22	1.32
χ^2^ p-value^b^			0.184		
One-way ANOVA p-value^c^					0.184
Individual socioeconomic status (SES)^a^					
Low	720	92.8%	7.2%	1.53	2.39
Middle	148	91.9%	8.1%	1.47	1.96
High	<10	∼	∼	∼	∼
Missing	53	92.5%	7.5%	1.34	1.63
χ^2^ p-value^b^			0.873		
One-way ANOVA p-value^c^					0.904
Neighborhood socioeconomic status (SES), quintile^e^					
Quintile 1 (Low)	313	91.1%	8.9%	1.52	2.20
Q2	326	92.3%	7.7%	1.58	2.41
Q3	197	94.4%	5.6%	1.41	2.31
Q4	87	96.6%	3.5%	1.36	2.06
Q5 (High)	<10	∼	∼	∼	∼
χ^2^ p-value^b^			0.42		
One-way ANOVA p-value^c^					0.851
Overall self-rated health, 10-point score^a^^,^^f^					
Quintile 1 (Low)	185	88.7%	11.3%	1.86	2.98
Q2	71	93.0%	7.0%	1.45	1.64
Q3	168	93.5%	6.5%	1.36	2.05
Q4	286	93.0%	7.0%	1.47	2.09
Q5 (High)	218	95.0%	5.0%	1.37	2.17
χ^2^ p-value^b^			0.179		
One-way ANOVA p-value^c^					0.191
Hypertension medication^a^					
No	608	92.6%	7.4%	1.53	2.46
Yes	320	92.8%	7.2%	1.45	1.89
χ^2^ P-value^b^			0.905		
One-way ANOVA p-value^c^					0.600
Previous cancer diagnosis^a^					
No	903	92.7%	7.3%	1.51	2.30
Yes	25	92.0%	8.0%	1.27	1.21
χ^2^ P-value^b^			0.896		
One-way ANOVA p-value^c^					0.602
Body mass index (BMI)^g^					
Underweight	40	97.5%	2.5%	1.32	0.91
Normal weight	322	92.9%	7.1%	1.48	2.21
Overweight	308	90.3%	9.7%	1.71	2.60
Obese	244	94.7%	5.3%	1.27	1.90
Missing	14	92.9%	7.4%	2.07	4.14
χ^2^ p-value^b^			0.243		
One-way ANOVA p-value^c^					0.181
Health insurance provider type^a^					
Medicaid	306	93.1%	6.9%	1.37	1.75
Uninsured	223	93.7%	6.3%	1.32	1.98
Other govt supported	273	91.9%	8.1%	1.65	2.61
Private or single payer	119	91.6%	8.4%	1.85	3.06
Missing	<10	∼	∼	∼	∼
χ^2^ p-value^b^			0.848		
One-way ANOVA p-value^c^					0.169
Last PSA test^a^					
Never	489	92.8%	7.2%	1.45	2.37
<1 year	162	92.6%	7.4%	1.56	1.99
1 to 5 years	165	90.9%	9.1%	1.87	2.62
>5 years ago	59	94.9%	5.1%	1.08	1.08
Unknown	53	94.3%	5.7%	1.15	1.96
χ^2^ p-value^b^			0.839		
One-way ANOVA p-value^c^					0.094
Last prostate exam^a^					
Never	492	93.3%	6.7%	1.51	2.47
<1 year	128	91.4%	8.6%	1.56	1.95
1 to 5 years	199	91.5%	8.5%	1.60	2.32
>5 years ago	109	93.6%	6.4%	1.26	1.54
χ^2^ p-value^b^			0.766		
One-way ANOVA p-value^c^					0.640
Visits to doctor in last 12 months^a^					
Quintile 1 (Low)	183	90.2%	9.8%	1.67	2.82
Q2	108	91.7%	8.3%	1.86	2.95
Q3	231	94.8%	5.2%	1.28	2.06
Q4	220	95.0%	5.0%	1.39	1.83
Q5 (High)	186	90.3%	9.7%	1.56	1.94
χ2 p-value^b^			0.154		
One-way ANOVA p-value^c^					0.159
Total	928	92.7%	7.3%	1.50	2.28

∼ Suppressed due to cell frequency <10.

a Based on self-report.

b χ^2^ P-values use binary PSA <4 vs. ≥4 ng/mL as the outcome.

c One-way ANOVA (analysis of variance) provided for continuous PSA as the outcome.

d Other tobacco use includes current regular use of E-cigarettes, cigars, pipes, snuff, chewing tobacco, and hookah.

e Quintiles of neighborhood-level contextual factors are modeled ordinally.

f Presented as quintiles for descriptive purposes only. Analyzed continuously.

g Based on direct measurement

Table 2 provides results for the association between cigarette smoking and PSA in fully adjusted logistic regression models with outcome of total PSA (≥4 ng/ mL [yes/ no]) and linear regression models with outcome of total PSA (continuous) in separate models specified for different categorizations of cigarette smoking: i) categorical never, 0–1, >1 pack-year; ii) categorical never, past, current; iii) categorical 0–19, ≥20 cigarettes per day; iv) continuous pack-years; and v) continuous cigarettes per day. Relative to never-smokers, AA men with > 1 pack-year of smoking experienced 4.34 times the odds of elevated PSA (odds ratio [OR] = 4.34; 95% confidence interval [CI] = 1.83 to 11.5; p = 0.002), after full adjustment. In separate models, we observed that past smokers experienced comparable risk estimates as those presented above for AA men with > 1 pack-year of smoking (OR = 4.58; 95% CI = 1.83 to 11.5; p = 0.0012; β=0.53; 95% CI = 0.02 to 1.04; p = 0.042), while no statistically significant differences in total PSA were observed for current smokers.

**Table 2:** Cigarette smoking and total serum prostate specific antigen (PSA) in fully adjusted^a^ regression models among 928 African American (AA) men in Chicago 2013–2018 – logistic regression models including odds ratio (OR) and 95% confidence interval (CI)(Model 1) with outcome of PSA >4 ng/mL and linear regression models includingβ coefficient and 95% CI (Model 2) with outcome of total PSA (continuous).

Participant Characteristics	Logistic regression models		Linear regression models	
	OR	(95% CI)	β	(95% CI)
Cigarette smoking history				
Never	1.00	(Reference)	0.00	(Reference)
0 to 1 pack-year	1.86	(0.84 to 4.12)	0.12	(−0.25 to 0.49)
>1 pack-year	4.34	(1.73 to 10.9)**	0.52	(0.01 to 1.04)*
Cigarette smoking history				
Never	1.00	(Reference)	0.00	(Reference)
Past	4.58	(1.83 to 11.5)*	0.53	(0.02 to 1.04)*
Current	1.83	(0.83 to 4.05)	0.12	(−0.26 to 0.49)
Cigarette smoking intensity				
0–19 cigarettes per day	1.00	(Reference)	0.00	(Reference)
20 or more cigarettes per day	2.00	(1.09 to 3.68)*	0.12	(−0.27 to 0.52)
Cigarette pack-years (continuous)	1.02	(1.00 to 1.04)	0.00	(−0.01 to 0.02)
Cigarettes per day (continuous)	1.02	(0.99 to 1.04)	0.00	(−0.02 to 0.02)

^b^ Type 3 Analysis of Effect on the relevant cigarette smoking variable; χ^2^ p-value for logistic regression models and F-ratio p-value for linear regression models.

a Adjusted for age (continuous), marijuana use, other current tobacco use including E-cigarettes, cigars, pipes, snuff, chewing tobacco, and hookah, marital status, individual and neighborhood socioeconomic status, self-reported health, previous cancer diagnosis, body mass index, hypertension medication (yes/no), health insurance type, timing of last prostate specific antigen test, timing of last prostate exam such as digital rectal exam, and visits to a doctor in the last 12 months (quintiles).

* p-value < 0.05.

** p-value <0.01.

Table 3 provides fully adjusted logistic regression and linear regression models with an outcome of elevated PSA and total PSA with a focus on behavioral factors, stratified by age. Cigarette smoking history was not associated with elevated PSA or mean PSA among 497 AA men aged 40 to < 55 years after full adjustment. However, among AA men aged ≥ 55 years, those with > 1 pack-year of smoking experienced a statistically significant five-fold increase in odds of elevated PSA relative to non-smokers (OR = 5.09; 95% CI = 1.57 to 16.6; p = 0.007), after full adjustment. After full adjustment for other factors examined, AA men aged ≥ 55 years who were current users of other tobacco products including e-cigarettes had a statistically significant increase in mean PSA of 1.20 ng/ mL (β = 1.20; 95% CI = 0.20 to 2.19; p = 0.019), which corresponded to a statistically non-significant 2.4-fold increase in odds of elevated PSA (OR = 2.38; 95% CI = 0.76 to 7.52; p = 0.138). No difference in odds of elevated or mean PSA were observed among current marijuana users aged 40 to 55 years, after adjusting for other factors. However, among AA men aged ≥ 55 years, current marijuana users experienced an approximate 73% reduction in the fully adjusted odds of elevated PSA relative to non-users (OR = 0.27; 95% CI = 0.08 to 0.96; p = 0.044), which corresponded to an approximate change in mean PSA = -0.69 ng/ mL (95% CI = -1.43 to 0.04; p = 0.062).

**Table 3:** Fully adjusted^a^ logistic regression models including odds ratio (OR) and 95% confidence interval (CI)(Model 1) with outcome of total serum prostate specific antigen (PSA) >4 ng/mL and linear regression withβ coefficient and 95% CI (Model 2) with outcome of total serum (continuous), stratified by age (y); Chicago 2013–2018.

Participant Characteristics	Model 1		Model 2	
	OR	(95% CI)	β	(95% CI)
Age				

Cigarette smoking history	40 to <55 years			
Never	1.00	(Reference)	0.00	(Reference)
0 to 1 pack-year	1.16	(0.29 to 4.72)	−0.11	(−0.49 to 0.28)
>1 pack-year	1.76	(0.18 to 16.8)	0.00	(−0.62 to 0.61)
Current marijuana use				
No	1.00	(Reference)	0.00	(Reference)
Yes	1.67	(0.47 to 5.92)	0.15	(−0.21 to 0.52)
Other current tobacco use^b^				
No	1.00	(Reference)	0.00	(Reference)
Yes	2.32	(0.56 to 9.59)	0.29	(−0.19 to 0.77)

Total	497			

Age	≥55 years			

Cigarette smoking history				
Never	1.00	(Reference)	0.00	(Reference)
0 to 1 pack-year	2.26	(0.76 to 6.73)	0.33	(−0.37 to 1.04)
>1 pack-year	5.09	(1.57 to 16.6)**	0.77	(−0.09 to 1.63)
Current marijuana use				
No	1.00	(Reference)	0.00	(Reference)
Yes	0.27	(0.08 to 0.96)*	−0.69	(−1.43 to 0.04)
Other current tobacco use^b^				
No	1.00	(Reference)	0.00	(Reference)
Yes	2.38	(0.76 to 7.52)	1.20	(0.20 to 2.19)*

Total	431			

Age	All ages			

Cigarette smoking history				
Never	1.00	(Reference)	0.00	(Reference)
0 to 1 pack-year	1.86	(0.84 to 4.12)	0.12	(−0.25 to 0.49)
>1 pack-year	4.34	(1.73 to 10.9)**	0.52	(0.01 to 1.04)*
Current marijuana use				
No	1.00	(Reference)	0.00	(Reference)
Yes	0.55	(0.25 to 1.22)	−0.19	(−0.56 to 0.17)
Other current tobacco use^b^				
No	1.00	(Reference)	0.00	(Reference)
Yes	1.99	(0.91 to 4.38)	0.63	(0.13 to 1.12)

Total	928			

a Adjusted for age (continuous), marital status, individual and neighborhood socioeconomic status, self-reported health, previous cancer diagnosis, body mass index, hypertension medication (yes/no), health insurance type, timing of last prostate specific antigen test, timing of last prostate exam such as digital rectal exam, and visits to a doctor in the last 12 months (quintiles).

b Other tobacco use includes current regular use of E-cigarettes, cigars, pipes, snuff, chewing tobacco, and hookah.

* p-value < 0.05

** p-value <0.01

Fully adjusted logistic regression and linear regression models with all co-variates are presented in Supplemental Table 3. Neither individual SES nor neighborhood SES were independently associated with odds of elevated PSA nor mean PSA (continuous). Incremental increases in self-reported overall health were associated with statistically significant 15% decreases in elevated PSA (OR = 0.85; 95% CI = 0.73 to 0.98; p = 0.029), after full adjustment. Relative to private insurance, statistically significant decreases in mean PSA were observed among AA men who reported Medicaid (β = -0.52; 95% CI = -1.03 to −0.01; p = 0.047) and uninsured (β = -0.59; 95% CI = -1.13 to −0.05; p = 0.032), after full adjustment. Visits to a doctor in the last 12 months were also associated with lower mean PSA, with a −0.13 ng/ mL change in mean PSA for each increase in quintile of visits to a doctor in the last 12 months (95% CI = -0.25 to 0.00; p = 0.043), after full adjustment. Non-substantive differences were observed across sub-items of type of visit to doctor’s offices.

Results from the sensitivity analysis of logistic regression models with separate cutoffs for PSA of 2.5, 4.0 and 10.0 ng/ mL are presented in Supplemental Table 4. We observed an effect modification for increasing serum PSA level and other current tobacco use among participants ≥ 55 years. In particular, AA men with other current tobacco use experienced 13.1 times the odds of 10.0 ng/ mL PSA as those without other tobacco use, after full adjustment (OR = 13.1; 95% CI = 2.09 to 82.3; p = 0.006). Robustness tests comparing the results from linear regression models with the mean PSA outcome to those with the natural-log transformed PSA outcome resulted in similar findings as reported here. Similarly, robustness tests with differing age cutoffs (<45 and ≥ 45, <50 and ≥ 50, and < 55 and 55 to 69 years), prior cancer (yes and no), and lifestyle factors resulted in similar findings as reported here.

## 6 Discussion

Little is known about factors that are associated with PSA levels, a marker of prostate cancer aggressiveness, among AA men who experience the greatest risk of prostate cancer mortality (Chornokur et al., 2011, Giovannucci et al., 2007). We examined associations between cigarette smoking history, other current tobacco use including e-cigarettes, and current marijuana use on PSA levels within a sample of AA men in Chicago. Among AA men ≥ 55 years, we found suggestive evidence that a history of heavy cigarette smoking was associated with increased odds of elevated PSA (≥4 ng/ mL), other current tobacco use was associated with continuous increases in PSA levels relative to non-users of other tobacco products, and current marijuana smoking was associated with a decreased odds of elevated PSA relative to non-marijuana smokers.

Cigarette smoking increases the risk of aggressive prostate cancer (Kenfield et al., 2011, Huncharek et al., 2010)and may increase incident prostate cancer among those who smoke the most (Huncharek et al., 2010). Temporal latency periods for cigarette smoking have been well-established for lung cancer trends (i.e., higher risk in older age groups) (Weiss, 1997 May) and may have relevance for prostate cancer risk. However, a National Health and Nutrition Examination Survey (NHANES) that included 18.6% AA men observed an inverse association between current or former smokers and PSA levels. The disparate findings may have been a result of different sample characteristics or study design. Importantly, our study comprised AA men with mean PSA level that exceeded each age-specific range reported in the NHANES study. Also, the NHANES study did not age-stratify findings, or examine pack-years of cigarette smoking to account for potential latency periods (Li et al., 2012). Although additional cross-sectional studies have observed an association between cigarette smoking and PSA levels, a pathophysiological mechanism of how cigarette smoking affects PSA levels is currently unknown (De Nunzio et al., 2019 Dec). Nevertheless, urologists are encouraged to recommend smoking cessation for their patients (Sosnowski et al., 2016).

To our knowledge, this is the first observational study to suggest that current marijuana use is inversely associated with serum PSA levels among men ≥ 55 years. Receptors for cannabinoids, the active component of marijuana, have increased expression in prostate cancer (Sarfaraz et al., 2005, Czifra et al., 2009), and additional evidence suggests dysregulation of these receptors is associated with prostate cancer (Chung et al., 2009, Diaz-Laviada, 2011). Our findings are consistent with basic science reports that a synthetic cannabinoid reduces PSA levels *in vitro* (Kenfield et al., 2011, Sarfaraz et al., 2005). Cannabinoids have additionally been shown to inhibit prostate cell growth *in vitro* (Sarfaraz et al., 2006, Roberto et al., 2019, Pacher, 2013) and to decrease pancreatic tumor growth *in vivo* (Carracedo et al., 2006). While there are some additional basic science reports that demonstrate that marijuana causes a reduction in testosterone in animal models, the effects of marijuana on human testosterone levels are less well understood (Banerjee et al., 2011, Wenger et al., 2001, List et al., 1977). Recent large epidemiologic studies of young men have demonstrated that there is no difference between testosterone levels in marijuana users compared to non-users (Gundersen et al., 2015, Thistle et al., 2017). However, an *in vitro* study suggested marijuana may decrease prostate responsiveness to testosterone by decreasing androgen receptor expression on prostate cells (Sosnowski et al., 2016). Multiple recent systematic reviews on the use of marijuana have however shown deleterious effects in testis size, semen parameters, (count, concentration, morphology, motility, and viability) and sexual function (Sarfaraz et al., 2005, Carvalho et al., 0000, Payne et al., 2019). While testosterone levels may be unaltered, marijuana’s effects on male glandular structures do provide insight that may in part explain the effects of current marijuana use on plasma concentrations of PSA in older men.

Separately, well-established evidence has identified that obesity contributes to decreases in PSA levels by increasing circulating plasma volumes (i.e., hemodilution of PSA), yet increases risk of aggressive prostate cancer (Bañez et al., 2007, Freedland and Aronson, 2004). This suggests that serum PSA may not be an appropriate biomarker for aggressive prostate cancer among obese men. It is also possible that serum PSA may not be a useful biomarker for aggressive prostate cancer among marijuana users. A recent clinical case series of 4,305 men diagnosed with localized prostate cancer did not demonstrate reduced prostate cancer aggressiveness among previous marijuana users (Huncharek et al., 2010). Marijuana use reducing PSA levels may reflect lower volume of benign tissue, less prostate cancer, or artificially lower PSA without impacting amount of benign of cancerous tissue. It is possible that a lower PSA cut-off for biopsy should be considered for current marijuana users, and perhaps magnetic resonance imaging may be a useful diagnostic tool for current marijuana users. Similar strategies have been suggested for clinical populations with low PSA levels that are not necessarily indicative of a low prostate cancer risk profile (Sanchis-Bonet et al., 2017).

We are unaware of previous studies that have examined the association between visits to doctor’s offices or clinics and PSA levels among men at high risk of advanced prostate cancer. In our study, we observed that AA men who reported having had more doctor’s office or clinic visits had a relatively small (<-0.14 ng/ mL) but statistically significant lower PSA levels in our multivariable models, including adjustment for BMI, health insurance type, and hypertension medication. Upon further investigation, we found that this finding persisted independent of usual healthcare setting, health insurance provider type, timing of last PSA test, and timing of last DRE.

It is unclear whether the findings presented in our study have clinical relevance for risk of prostate cancer, since PSA ≥ 4.0 ng/ mL has a relatively low sensitivity but remains the most common threshold for recommending further imaging or a biopsy. Findings from a trial of 18,882 healthy men 55 years of age or older reported that only 20% of men with PSA ≥ 4.0 ng/mL actually had the disease, and 6% of men who do not have prostate cancer falsely tested positive at this threshold (Ankerst and Thompson, 2006). There are other urine and serum biomarkers that are used as serial tests that are able to improve test accuracy (e.g. 4 K score, Prostate Health Index, Select MDx, etc). We also reported findings for cutoffs of 2.0 and 10.0 ng/ mL. Future work in prospective cohort studies with information on prostate cancer outcomes in diverse patient populations are critical to assess whether the factors examined in our study may contribute to increased prostate cancer risk in AA men.

Our study was strengthened by the relatively large sample of AA men–a group traditionally under-represented in research involving PSA measurements and more likely than other populations to experience disparities in prostate cancer aggressiveness and mortality. Additionally, we tailored recruitment strategies to AA participants, including concordance of interviewer race/ ethnicity and provision of bio-specimen education to community members.

Our study was limited by our inability to screen for prostate cancer. Furthermore, our study was comprised of relatively few participants with elevated PSA, which limited statistical power. An additional limitation of our study was that our results were based on a single PSA measure at one point in time. PSA Measures can fluctuate and the PSA on a given day may not be representative of a participant’s average PSA. Additionally, a single PSA test lacks potentially important information such as PSA kinetics (PSA velocity and doubling time) and free-to-total PSA ratio, which are potentially important predictors of prostate cancer aggressiveness (Catalona et al., 2000, Carter et al., 1992, Vickers and Brewster, 2012). Our study was further subject to limitations common to observational studies. Generalizability to AA men who are not predominantly of low SES, residing in low SES neighborhoods, is unclear. Findings presented in our study may have also been due to residual confounding. Further, our findings relied on participant self-reporting, which may have been prone to recall bias. We minimized the threat of recall bias by study design features to maximize interviewer rapport – specifically training race-concordant interviewers with previous experience in low SES communities. Interestingly, 20.2% of our sample self-reported marijuana use, which was illegal in Chicago at the time of the study, except for medical dispensaries. This improves our confidence in self-report for our sample. Nevertheless, specific measures with longer look-back periods may have been particularly subject to recall bias, including healthcare utilization in the last 12 months, medical history, and medications.

We were further unable to account for unobserved characteristics such as plasma volume, sexual behavior and ejaculation patterns, sleep patterns, and genetic variants that may be associated with both PSA levels and the factors examined in our study (Bañez et al., 2007, Tarhan et al., 2016, Grubb et al., 2009, Singer and DiPaola, 2013, Werny et al., 2007). Additionally, marijuana smoking was only captured as binary outcome by current use status. Given the heterogeneity of the chemical composition of different types of marijuana, the concentration of the marijuana dose, and the frequency and chronicity of use potentially influencing PSA levels, future prospective studies will be required to determine if these factors also influence PSA levels. Specifically, our study was unable to assess the effect of timing between marijuana use or tobacco use and serum PSA levels. Future research may assess whether PSA temporarily lowers after marijuana use and then stabilizes, or whether there is a threshold of marijuana use over time that is required before PSA levels are impacted. Furthermore, blunts with mixed tobacco and marijuana, which may be common in Chicago, were not separately assessed. Our study also suffered from limited statistical power to detect modest associations or to examine tobacco products separately such as e-cigarettes.

## 7 Conclusion

Generating knowledge about vulnerable populations is an important priority for reducing social inequities in cancer (Vaccarella et al., 2018). We found suggestive evidence that cigarette smoking history and other current tobacco use may be associated with serum PSA in older AA men, whereas marijuana use may be inversely associated with serum PSA in older AA men. Future studies with cancer outcomes data will be highly relevant for better understanding risk of aggressive prostate cancer among AA men, as well as for targeting communities and individuals who may be more likely to experience benefit from PSA testing. In particular, future work may elucidate whether exposure to pack-years of cigarette smoking is associated with plasma concentrations of PSA (and link with biopsy outcomes), particularly in populations of men at high risk of aggressive prostate cancer. Furthermore, future work examining e-cigarettes and marijuana are warranted. Specifically, it is unclear whether PSA is an accurate biomarker of prostate cancer aggressiveness among older current marijuana users.

## Declaration of Competing Interest

The authors declare that they have no known competing financial interests or personal relationships that could have appeared to influence the work reported in this paper.
