# Supplementary material for: Tobacco and marijuana use and their association with serum prostate-specific antigen levels among African American men in Chicago
Source: Prev Med Rep. 2020 Aug 11;20:101174. doi: 10.1016/j.pmedr.2020.101174 (PMC7566952; doi:10.1016/j.pmedr.2020.101174)
Supplement: Supplementary data 1 [file mmc1.docx]

**Supplemental Table 1.** Participant characteristics and mean prostate-specific antigen (PSA; ng/ mL) level among 497 African American (AA) men aged 40 to <55 years, Chicago 2013-2018

| **Participant Characteristics** | **n** | **Dichotomous** | |  | **Continuous** | |
| --- | --- | --- | --- | --- | --- | --- |
|  |  | **PSA <4**  **(row %)** | **PSA ≥4**  **(row %)** |  | **Mean PSA** | **St. Dev.** |
| **Behavioral factors** |  |  |  |  |  |  |
| Cigarette smoking history^a^ |  |  |  |  |  |  |
| Never | 105 | 96.2% | 3.8% |  | 1.18 | 1.87 |
| 0 to 1 pack-year | 349 | 95.7% | 4.3% |  | 1.18 | 1.72 |
| >1 pack-year | 43 | 95.4% | 4.6% |  | 1.02 | 1.08 |
| χ^2^ p-value^b^ |  |  | 0.966 |  |  |  |
| One-way ANOVA p-value^c^ |  |  |  |  |  | 0.850 |
| Other current tobacco use^a,d^ |  |  |  |  |  |  |
| No | 440 | 96.1% | 3.9% |  | 1.14 | 1.57 |
| Yes | 57 | 93.0% | 7.0% |  | 1.36 | 2.52 |
| χ^2^ P-value^b^ |  |  | 0.265 |  |  |  |
| One-way ANOVA p-value^c^ |  |  |  |  |  | 0.355 |
| Current marijuana use |  |  |  |  |  |  |
| No | 382 | 95.8% | 4.2% |  | 1.16 | 1.84 |
| Yes | 115 | 95.7% | 4.3% |  | 1.18 | 1.16 |
| χ^2^ P-value^b^ |  |  | 0.941 |  |  |  |
| One-way ANOVA p-value^c^ |  |  |  |  |  | 0.904 |
| **Co-variates** |  |  |  |  |  |  |
| Age (years)^a^ |  |  |  |  |  |  |
| 40 to <45 | 108 | 98.2% | 1.8% |  | 0.99 | 0.94 |
| 45 to <50 | 176 | 97.2% | 2.8% |  | 0.97 | 1.06 |
| 50 to <55 | 213 | 93.4% | 6.6% |  | 1.42 | 2.30 |
| χ^2^ p-value^b^ |  |  | 0.072 |  |  |  |
| One-way ANOVA p-value^c^ |  |  |  |  |  | 0.018 |
| Marital status^a^ |  |  |  |  |  |  |
| Single, never married | 277 | 95.7% | 4.3% |  | 1.10 | 1.22 |
| Married | 77 | 97.4% | 2.6% |  | 1.09 | 1.37 |
| Living with partner | 28 | 96.4% | 3.6% |  | 1.25 | 0.86 |
| Separated | 37 | 97.3% | 2.7% |  | 1.63 | 3.36 |
| Divorced | 70 | 92.9% | 7.1% |  | 0.94 | 0.48 |
| Widowed | <10 | ~ | ~ |  | ~ | ~ |
| χ^2^ p-value^b^ |  |  | 0.860 |  |  |  |
| One-way ANOVA p-value^c^ |  |  |  |  |  | 0.343 |
| Individual socioeconomic status (SES)^a^ |  |  |  |  |  |  |
| Low | 380 | 96.1% | 3.9% |  | 1.16 | 1.80 |
| Middle | 87 | 95.4% | 4.6% |  | 1.22 | 1.28 |
| High | <10 | ~ | ~ |  | ~ | ~ |
| Missing | 26 | 92.3% | 7.7% |  | 1.15 | 1.77 |
| χ^2^ p-value^b^ |  |  | 0.789 |  |  |  |
| One-way ANOVA p-value^c^ |  |  |  |  |  | 0.961 |
| Neighborhood socioeconomic status (SES), quintile^e^ | | |  |  |  |  |
| Quintile 1 (Low) | 179 | 94.4% | 5.6% |  | 1.23 | 1.68 |
| Q2 | 167 | 95.8% | 4.2% |  | 1.28 | 2.20 |
| Q3 | 109 | 97.3% | 2.8% |  | 0.96 | 0.90 |
| Q4 | 40 | 100.0% | 0.0% |  | 0.89 | 0.76 |
| Q5 (High) | <10 | ~ | ~ |  | ~ | ~ |
| χ^2^ p-value^b^ |  |  | 0.009 |  |  |  |
| One-way ANOVA p-value^c^ |  |  |  |  |  | 0.145 |

**Supplemental Table 1 continued.**

| **Participant Characteristics** | |  | | | **n** | | **Dichotomous** | | |  | **Continuous** | | | |
| --- | --- | --- | --- | --- | --- | --- | --- | --- | --- | --- | --- | --- | --- | --- |
|  |  |  |  |  |  |  | **PSA <4**  **(row %)** | **PSA ≥4**  **(row %)** | |  | **Mean PSA** | | **St. Dev.** | |
| Overall self-rated health, 10-point score^a,f^ | | | | |  | |  |  | |  |  | |  | |
| Quintile 1 (Low) | | |  | | 74 | | 91.9% | 8.1% | |  | 1.42 | | 2.32 | |
| Q2 | | |  | | 34 | | 100.0% | 0.0% | |  | 0.88 | | 0.68 | |
| Q3 | | |  | | 86 | | 100.0% | 0.0% | |  | 0.81 | | 0.63 | |
| Q4 | | |  | | 164 | | 92.7% | 7.3% | |  | 1.30 | | 1.54 | |
| Q5 (High) | | |  | | 139 | | 97.8% | 2.2% | |  | 1.16 | | 2.07 | |
| χ^2^ p-value^b^ | | |  | |  | |  | 0.010 | |  |  | |  | |
| One-way ANOVA p-value^c^ | | |  | |  | |  |  | |  |  | | 0.115 | |
| Hypertension medication^a^ | | |  | |  | |  |  | |  |  | |  | |
| No | | |  | | 382 | | 95.8% | 4.2% | |  | 1.19 | | 1.81 | |
| Yes | | |  | | 115 | | 95.7% | 4.3% | |  | 1.08 | | 1.30 | |
| χ^2^ P-value^b^ | | |  | |  | |  | 0.941 | |  |  | |  | |
| One-way ANOVA p-value^c^ | | |  | |  | |  |  | |  |  | | 0.552 | |
| Previous cancer diagnosis^a^ | | |  | |  | |  |  | |  |  | |  | |
| No | | |  | | 489 | | 95.9% | 4.1% | |  | 1.16 | | 1.71 | |
| Yes | | |  | | <10 | | ~ | ~ | |  | ~ | | ~ | |
| χ^2^ P-value^b^ | | |  | |  | |  | 0.241 | |  |  | | 0.468 | |
| One-way ANOVA p-value^c^ | | |  | |  | |  |  | |  |  | |  | |
| Body mass index (BMI)^g^ | | |  | |  | |  |  | |  |  | |  | |
| Underweight | | |  | | 15 | | 97.5% | 2.5% | |  | 1.38 | | 1.03 | |
| Normal weight | | |  | | 176 | | 92.9% | 7.1% | |  | 1.18 | | 2.04 | |
| Overweight | | |  | | 168 | | 90.3% | 9.7% | |  | 1.41 | | 1.86 | |
| Obese | | |  | | 131 | | 94.7% | 5.3% | |  | 0.83 | | 0.86 | |
| Missing | | |  | | <10 | | ~ | ~ | |  | ~ | | ~ | |
| χ^2^ p-value^b^ | | |  | |  | |  | 0.044 | |  |  | |  | |
| One-way ANOVA p-value^c^ | | |  | |  | |  |  | |  |  | | 0.061 | |
| Health insurance provider type^a^ | | |  | |  | |  |  | |  |  | |  | |
| Medicaid | | |  | | 161 | | 96.3% | 3.7% | |  | 1.13 | | 1.26 | |
| Uninsured | | |  | | 163 | | 96.3% | 3.7% | |  | 1.13 | | 1.70 | |
| Other govt supported | | |  | | 103 | | 96.1% | 3.9% | |  | 1.02 | | 0.99 | |
| Private or single payer | | |  | | 64 | | 92.2% | 7.8% | |  | 1.62 | | 3.09 | |
| Missing | | |  | | <10 | | ~ | ~ | |  | ~ | | ~ | |
| χ^2^ p-value^b^ | | |  | |  | |  | 0.636 | |  |  | |  | |
| One-way ANOVA p-value^c^ | | |  | |  | |  |  | |  |  | | 0.243 | |
| Last PSA test^a^ | | |  | |  | |  |  | |  |  | |  | |
| Never | | |  | | 318 | | 94.7% | 5.3% | |  | 1.26 | | 2.01 | |
| <1 year | | |  | | 58 | | 96.6% | 3.4% | |  | 0.95 | | 0.94 | |
| 1 to 5 years | | |  | | 67 | | 98.5% | 1.5% | |  | 1.18 | | 0.96 | |
| >5 years ago | | |  | | 30 | | 96.7% | 3.3% | |  | 0.95 | | 1.03 | |
| Unknown | | |  | | 24 | | 100.0% | 0.0% | |  | 0.64 | | 0.39 | |
| χ^2^ p-value^b^ | | |  | |  | |  | 0.489 | |  |  | |  | |
| One-way ANOVA p-value^c^ | | |  | |  | |  |  | |  |  | | 0.319 | |
| Last prostate exam^a^ | | |  | |  | |  |  | |  |  | |  | |
| Never | | |  | | 313 | | 96.2% | 3.8% | |  | 1.23 | | 1.94 | |
| <1 year | | |  | | 54 | | 90.7% | 9.3% | |  | 1.33 | | 1.42 | |
| 1 to 5 years | | |  | | 77 | | 94.8% | 5.2% | |  | 1.05 | | 1.33 | |
| >5 years ago | | |  | | 53 | | 100.0% | 0.0% | |  | 0.78 | | 0.65 | |
| χ^2^ p-value^b^ | | |  | |  | |  | 0.111 | |  |  | |  | |
| One-way ANOVA p-value^c^ | | |  | |  | |  |  | |  |  | | 0.261 | |
|  | | |  | |  |  | |  | |  |  | |  | |
|  | | |  | |  |  | |  | |  |  | |  | |
|  | | |  | |  |  | |  | |  |  | |  | |
| **Supplemental Table 1 continued.** | | | | | |  | |  | |  |  | |  | |
| **Participant Characteristics** | | |  | | **n** | **Dichotomous** | | | |  | **Continuous** | | | |
|  |  |  |  |  |  | **PSA <4**  **(row %)** | | **PSA ≥4**  **(row %)** | |  | **Mean PSA** | | **St. Dev.** | |
| Visits to doctor in last 12 months^a^ | | |  | |  |  | |  | |  |  | |  | |
| Quintile 1 (Low) | | |  | | 123 | 091.9% | | 8.1% | |  | 1.56 | | 2.84 | |
| Q2 | | |  | | 64 | 096.9% | | 3.1% | |  | 1.16 | | 1.29 | |
| Q3 | | |  | | 132 | 097.0% | | 3.0% | |  | 1.05 | | 1.24 | |
| Q4 | | |  | | 98 | 100.0% | | 0.0% | |  | 0.89 | | 0.61 | |
| Q5 (High) | | |  | | 80 | 093.8% | | 6.2% | |  | 1.08 | | 1.07 | |
| χ2 p-value^b^ | | |  | |  |  | | 0.034 | |  |  | |  | |
| One-way ANOVA p-value^c^ | | |  | |  |  | |  | |  |  | | 0.042 | |
| Total | | |  | | 497 | 095.8% | | 4.2% | |  | 1.17 | | 1.71 | |
| ^a^ Based on self-report. | | | | | | | | | | | | | | |
| ^b^ χ^2^ P-values use binary PSA <4 vs. ≥4 ng/ mL as the outcome. | | | | | | | | | | | | | | |
| ^c^ One-way ANOVA (analysis of variance) provided for continuous PSA as the outcome. | | | | | | | | | | | | | | |
| ^d^ Other tobacco use includes current regular use of E-cigarettes, cigars, pipes, snuff, chewing tobacco, and hookah. | | | | | | | | | | | | | | |
| ^e^ Quintiles of neighborhood-level contextual factors are modeled ordinally. | | | | | | | | | | | | | | |
| ^f^ Presented as quintiles for descriptive purposes only. Analyzed continuously. | | | | | | | | | | | | | | |
| ^g^ Based on direct measurement | | | | | | | | | | | | | | |
| ~ Suppressed due to cell frequency <10 | | | | | | | | | | | | | | |

**Supplemental Table 2.** Participant characteristics and mean prostate-specific antigen (PSA; ng/ mL) level among 431 African American (AA) men aged ≥55 years, Chicago 2013-2018

| **Participant Characteristics** | **n** | **Dichotomous** | |  | **Continuous** | |
| --- | --- | --- | --- | --- | --- | --- |
|  |  | **PSA <4**  **(row %)** | **PSA ≥4**  **(row %)** |  | **Mean PSA** | **St. Dev.** |
| **Behavioral factors** |  |  |  |  |  |  |
| Cigarette smoking history^a^ |  |  |  |  |  |  |
| Never | 93 | 94.6% | 05.4% |  | 1.64 | 2.19 |
| 0 to 1 pack-year | 256 | 89.5% | 10.5% |  | 1.86 | 2.82 |
| >1 pack-year | 82 | 81.7% | 18.3% |  | 2.29 | 3.08 |
| χ^2^ p-value^b^ |  |  | 0.023 |  |  |  |
| One-way ANOVA p-value^c^ |  |  |  |  |  | 0.282 |
| Other current tobacco use^a,d^ |  |  |  |  |  |  |
| No | 397 | 89.7% | 10.3% |  | 1.80 | 2.48 |
| Yes | 34 | 82.4% | 17.6% |  | 3.00 | 4.82 |
| χ^2^ P-value^b^ |  |  | 0.189 |  |  |  |
| One-way ANOVA p-value^c^ |  |  |  |  |  | 0.014 |
| Current marijuana use |  |  |  |  |  |  |
| No | 359 | 87.7% | 12.3% |  | 1.99 | 2.78 |
| Yes | 72 | 95.8% | 04.2% |  | 1.42 | 2.58 |
| χ^2^ P-value^b^ |  |  | 0.044 |  |  |  |
| One-way ANOVA p-value^c^ |  |  |  |  |  | 0.105 |
| **Co-variates** |  |  |  |  |  |  |
| Age (years)^a^ |  |  |  |  |  |  |
| 55 to <60 | 196 | 90.3% | 09.7% |  | 1.69 | 2.44 |
| ≥60 | 235 | 88.1% | 11.9% |  | 2.07 | 2.98 |
| χ^2^ p-value^b^ |  |  | 0.461 |  |  |  |
| One-way ANOVA p-value^c^ |  |  |  |  |  | 0.154 |
| Marital status^a^ |  |  |  |  |  |  |
| Single, never married | 173 | 93.1% | 06.9% |  | 1.72 | 2.92 |
| Married | 81 | 87.7% | 12.3% |  | 2.03 | 2.71 |
| Living with partner | 25 | 84.0% | 16.0% |  | 2.20 | 2.34 |
| Separated | 37 | 86.5% | 13.5% |  | 2.05 | 3.28 |
| Divorced | 86 | 83.7% | 16.3% |  | 2.17 | 2.65 |
| Widowed | 27 | 92.6% | 07.4% |  | 1.29 | 1.46 |
| χ^2^ p-value^b^ |  |  | 0.317 |  |  |  |
| One-way ANOVA p-value^c^ |  |  |  |  |  | 0.749 |
| Individual socioeconomic status (SES)^a^ |  |  |  |  |  |  |
| Low | 340 | 89.1% | 10.9% |  | 1.94 | 2.86 |
| Middle | 61 | 86.9% | 13.1% |  | 1.82 | 2.61 |
| High | <10 | ~ | ~ |  | ~ | ~ |
| Missing | 27 | 92.6% | 07.4% |  | 1.52 | 1.49 |
| χ^2^ p-value^b^ |  |  | 0.798 |  |  |  |
| One-way ANOVA p-value^c^ |  |  |  |  |  | 0.877 |
| Neighborhood socioeconomic status (SES), quintile^e^ | | |  |  |  |  |
| Quintile 1 (Low) | 134 | 86.6% | 13.4% |  | 1.91 | 2.70 |
| Q2 | 159 | 88.7% | 11.3% |  | 1.90 | 2.57 |
| Q3 | 88 | 90.9% | 9.1% |  | 1.96 | 3.23 |
| Q4 | 47 | 93.6% | 6.4% |  | 1.76 | 2.67 |
| Q5 (High) | <10 | ~ | ~ |  | ~ | ~ |
| χ^2^ p-value^b^ |  |  | 0.633 |  |  |  |
| One-way ANOVA p-value^c^ |  |  |  |  |  | 0.986 |

**Supplemental Table 2 continued.**

| **Participant Characteristics** | |  | | | **n** | | **Dichotomous** | | |  | **Continuous** | | | |
| --- | --- | --- | --- | --- | --- | --- | --- | --- | --- | --- | --- | --- | --- | --- |
|  |  |  |  |  |  |  | **PSA <4**  **(row %)** | **PSA ≥4**  **(row %)** | |  | **Mean PSA** | | **St. Dev.** | |
| Overall self-rated health, 10-point score^a,f^ | | | | |  | |  |  | |  |  | |  | |
| Quintile 1 (Low) | | |  | | 111 | | 86.5% | 13.5% | |  | 2.16 | | 3.32 | |
| Q2 | | |  | | 37 | | 86.5% | 13.5% | |  | 1.97 | | 2.05 | |
| Q3 | | |  | | 82 | | 86.6% | 13.4% | |  | 1.94 | | 2.76 | |
| Q4 | | |  | | 122 | | 93.4% | 06.6% | |  | 1.70 | | 2.63 | |
| Q5 (High) | | |  | | 79 | | 89.9% | 10.1% | |  | 1.74 | | 2.31 | |
| χ^2^ p-value^b^ | | |  | |  | |  | 0.407 | |  |  | |  | |
| One-way ANOVA p-value^c^ | | |  | |  | |  |  | |  |  | | 0.750 | |
| Hypertension medication^a^ | | |  | |  | |  |  | |  |  | |  | |
| No | | |  | | 226 | | 87.2% | 12.8% | |  | 2.11 | | 3.20 | |
| Yes | | |  | | 205 | | 91.2% | 08.8% | |  | 1.66 | | 2.12 | |
| χ^2^ P-value^b^ | | |  | |  | |  | 0.178 | |  |  | |  | |
| One-way ANOVA p-value^c^ | | |  | |  | |  |  | |  |  | | 0.09 | |
| Previous cancer diagnosis^a^ | | |  | |  | |  |  | |  |  | |  | |
| No | | |  | | 414 | | 88.9% | 11.1% | |  | 1.93 | | 2.79 | |
| Yes | | |  | | 17 | | 94.1% | 05.9% | |  | 1.12 | | 1.13 | |
| χ^2^ P-value^b^ | | |  | |  | |  | 0.498 | |  |  | |  | |
| One-way ANOVA p-value^c^ | | |  | |  | |  |  | |  |  | | 0.23 | |
| Body mass index (BMI)^g^ | | |  | |  | |  |  | |  |  | |  | |
| Underweight | | |  | | 25 | | 100.0% | 00.0% | |  | 1.28 | | 0.86 | |
| Normal weight | | |  | | 146 | | 88.4% | 11.6% | |  | 1.85 | | 2.36 | |
| Overweight | | |  | | 140 | | 87.9% | 12.1% | |  | 2.08 | | 3.25 | |
| Obese | | |  | | 113 | | 89.4% | 14.3% | |  | 1.79 | | 2.55 | |
| Missing | | |  | | <10 | | ~ | ~ | |  | ~ | | ~ | |
| χ^2^ p-value^b^ | | |  | |  | |  | 0.485 | |  |  | |  | |
| One-way ANOVA p-value^c^ | | |  | |  | |  |  | |  |  | | 0.438 | |
| Health insurance provider type^a^ | | |  | |  | |  |  | |  |  | |  | |
| Medicaid | | |  | | 145 | | 89.7% | 13.3% | |  | 1.65 | | 2.14 | |
| Uninsured | | |  | | 60 | | 86.7% | 10.3% | |  | 1.82 | | 2.55 | |
| Other govt supported | | |  | | 170 | | 89.4% | 10.6% | |  | 2.04 | | 3.16 | |
| Private or single payer | | |  | | 55 | | 90.9% | 09.1% | |  | 2.13 | | 3.03 | |
| Missing | | |  | | <10 | | ~ | ~ | |  | ~ | | ~ | |
| χ^2^ p-value^b^ | | |  | |  | |  | 0.067 | |  |  | |  | |
| One-way ANOVA p-value^c^ | | |  | |  | |  |  | |  |  | |  | |
| Last PSA test^a^ | | |  | |  | |  |  | |  |  | |  | |
| Never | | |  | | 171 | | 89.5% | 10.5% | |  | 1.81 | | 2.90 | |
| <1 year | | |  | | 104 | | 90.4% | 09.6% | |  | 1.89 | | 2.32 | |
| 1 to 5 years | | |  | | 98 | | 85.7% | 14.3% | |  | 2.34 | | 3.22 | |
| >5 years ago | | |  | | 29 | | 93.1% | 06.9% | |  | 1.21 | | 1.13 | |
| Unknown | | |  | | 29 | | 89.7% | 10.3% | |  | 1.58 | | 2.57 | |
| χ^2^ p-value^b^ | | |  | |  | |  | 0.764 | |  |  | |  | |
| One-way ANOVA p-value^c^ | | |  | |  | |  |  | |  |  | | 0.292 | |
| Last prostate exam^a^ | | |  | |  | |  |  | |  |  | |  | |
| Never | | |  | | 179 | | 88.3% | 11.7% | |  | 1.98 | | 3.15 | |
| <1 year | | |  | | 74 | | 91.9% | 08.1% | |  | 1.73 | | 2.26 | |
| 1 to 5 years | | |  | | 122 | | 89.3% | 10.7% | |  | 1.95 | | 2.72 | |
| >5 years ago | | |  | | 56 | | 87.5% | 12.5% | |  | 1.71 | | 1.96 | |
| χ^2^ p-value^b^ | | |  | |  | |  | 0.831 | |  |  | |  | |
| One-way ANOVA p-value^c^ | | |  | |  | |  |  | |  |  | | 0.860 | |
|  | | |  | |  |  | |  | |  |  | |  | |
|  | | |  | |  |  | |  | |  |  | |  | |
|  | | |  | |  |  | |  | |  |  | |  | |
| **Supplemental Table 2 continued.** | | | | | |  | |  | |  |  | |  | |
| **Participant Characteristics** | | |  | | **n** | **Dichotomous** | | | |  | **Continuous** | | | |
|  |  |  |  |  |  | **PSA <4**  **(row %)** | | **PSA ≥4**  **(row %)** | |  | **Mean PSA** | | **St. Dev.** | |
| Visits to doctor in last 12 months^a^ | | |  | |  |  | |  | |  |  | |  | |
| Quintile 1 (Low) | | |  | | 60 | 86.7% | | 13.3% | |  | 1.88 | | 2.80 | |
| Q2 | | |  | | 44 | 84.1% | | 15.9% | |  | 2.88 | | 4.18 | |
| Q3 | | |  | | 99 | 91.9% | | 08.1% | |  | 1.58 | | 2.78 | |
| Q4 | | |  | | 122 | 91.0% | | 09.0% | |  | 1.79 | | 2.32 | |
| Q5 (High) | | |  | | 106 | 87.7% | | 12.3% | |  | 1.92 | | 2.33 | |
| χ2 p-value^b^ | | |  | |  |  | | 0.565 | |  |  | |  | |
| One-way ANOVA p-value^c^ | | |  | |  |  | |  | |  |  | | 0.123 | |
| Total | | |  | | 431 | 89.1% | | 09.1% | |  | 1.90 | | 2.75 | |
| ^a^ Based on self-report. | | | | | | | | | | | | | | |
| ^b^ χ^2^ P-values use binary PSA <4 vs. ≥4 ng/ mL as the outcome. | | | | | | | | | | | | | | |
| ^c^ One-way ANOVA (analysis of variance) provided for continuous PSA as the outcome. | | | | | | | | | | | | | | |
| ^d^ Other tobacco use includes current regular use of E-cigarettes, cigars, pipes, snuff, chewing tobacco, and hookah. | | | | | | | | | | | | | | |
| ^e^ Quintiles of neighborhood-level contextual factors are modeled ordinally. | | | | | | | | | | | | | | |
| ^f^ Presented as quintiles for descriptive purposes only. Analyzed continuously. | | | | | | | | | | | | | | |
| ^g^ Based on direct measurement | | | | | | | | | | | | | | |
| ~ Suppressed due to cell frequency <10 | | | | | | | | | | | | | | |

**Supplemental Table 3.** Fully adjusted logistic regression including odds ratios (OR) and 95% confidence intervals (CIs) with outcome of total serum prostate specific antigen (PSA) >4 ng/ mL (Model 1) and linear regression models including $\beta$ coefficient and 95% CIs with outcome of total serum PSA (continuous) among 928 African American (AA) men in Chicago 2013 to 2018.

| **Participant Characteristics** | **Model 1** | |  | **Model 2** | |
| --- | --- | --- | --- | --- | --- |
|  | **OR** | **(95% CI)** |  | $\boldsymbol{\beta}$ | **(95% CI)** |
| **Lifestyle characteristics** |  |  |  |  |  |
| Current marijuana use |  |  |  |  |  |
| No | 1.00 | (Reference) |  | 0.00 | (Reference) |
| Yes | 0.55 | (0.25 to 1.22) |  | -0.19 | (-0.56 to 0.17) |
| Other current tobacco use^a^ |  |  |  |  |  |
| No | 1.00 | (Reference) |  | 0.00 | (Reference) |
| Yes | 1.99 | (0.91 to 4.38) |  | 0.63 | (0.13 to 1.12) |
| Cigarette smoking history |  |  |  |  |  |
| Never | 1.00 | (Reference) |  | 0.00 | (Reference) |
| 0 to 1 pack-year | 1.86 | (0.84 to 4.12) |  | 0.12 | (-0.25 to 0.49) |
| >1 pack-year | 4.34 | (1.73 to 10.9) |  | 0.52 | (0.01 to 1.04) |
| **Co-variates** |  |  |  |  |  |
| Age (years) | 1.09 | (1.05 to 1.13) |  | 0.06 | (0.04 to 0.08) |
| Marital status |  |  |  |  |  |
| Married | 1.00 | (Reference) |  | 0.00 | (Reference) |
| Single, never married | 0.93 | (0.41 to 2.10) |  | -0.06 | (-0.49 to 0.36) |
| Divorced | 1.84 | (0.78 to 4.30) |  | 0.36 | (-0.15 to 0.87) |
| Living with partner | 1.49 | (0.46 to 4.88) |  | 0.20 | (-0.51 to 0.90) |
| Separated | 1.03 | (0.34 to 3.11) |  | 0.00 | (-0.62 to 0.65) |
| Widowed | 0.49 | (0.10 to 2.52) |  | -0.67 | (-1.51 to 0.17) |
| Individual SES |  |  |  |  |  |
| Low | 1.00 | (Reference) |  | 0.00 | (Reference) |
| Middle or high | 1.28 | (0.66 to 2.49) |  | -0.16 | (-0.53 to 0.21) |
| Neighborhood socioeconomic status (SES) | 1.30 | (0.98 to 1.72) |  | 0.07 | (-0.07 to 0.22) |
| Overall health, 10-point score | 0.85 | (0.73 to 0.98) |  | -0.07 | (-0.16 to 0.01) |
| Hypertension medication |  |  |  |  |  |
| No | 1.00 | (Reference) |  | 0.00 | (Reference) |
| Yes | 0.64 | (0.33 to 1.24) |  | -0.36 | (-0.82 to -0.14) |
| Previous cancer diagnosis |  |  |  |  |  |
| No | 1.00 | (Reference) |  | 0.00 | (Reference) |
| Yes | 0.78 | (0.16 to 3.79) |  | -0.44 | (-1.35 to 0.46) |
| Body mass index (BMI) |  |  |  |  |  |
| Normal weight | 1.00 | (Reference) |  | 0.00 | (Reference) |
| Underweight | 0.25 | (0.03 to 1.99) |  | -0.37 | (-1.11 to 0.37) |
| Overweight | 1.62 | (0.88 to 3.00) |  | 0.27 | (-0.09 to 0.62) |
| Obese | 0.66 | (0.30 to 1.45) |  | -0.20 | (-0.59 to 0.20) |
| Missing | 1.20 | (0.12 to 11.7) |  | 0.74 | (-0.48 to 1.96) |
| Health insurance provider type |  |  |  |  |  |
| Private or single payer | 1.00 | (Reference) |  | 0.00 | (Reference) |
| Uninsured | 0.78 | (0.30 to 2.03) |  | -0.59 | (-1.13 to -0.05) |
| Medicaid | 0.90 | (0.37 to 2.18) |  | -0.52 | (-1.03 to -0.01) |
| Other government supported | 0.70 | (0.29 to 1.70) |  | -0.48 | (-0.99 to 0.04) |
| Missing | 3.18 | (0.30 to 34.1) |  | -0.25 | (-1.96 to 1.46) |

**Supplemental Table 3 continued.**

| **Participant Characteristics** | **Model 1** | |  | **Model 2** | |
| --- | --- | --- | --- | --- | --- |
|  | **OR** | **(95% CI)** |  | $\boldsymbol{\beta}$ | **(95% CI)** |
| Last PSA test |  |  |  |  |  |
| Never | 1.00 | (Reference) |  | 0.00 | (Reference) |
| <1 year | 0.61 | (0.23 to 1.59) |  | 0.00 | (-0.50 to 0.50) |
| 1 to 5 years | 0.82 | (0.37 to 1.83) |  | 0.24 | (-0.22 to 0.70) |
| >5 years | 0.66 | (0.16 to 2.79) |  | -0.27 | (-1.01 to 0.47) |
| Don’t know | 0.51 | (0.14 to 1.90) |  | -0.44 | (-1.10 to 0.21) |
| Last prostate exam such as digital rectal exam (DRE) |  |  |  |  |  |
| Never | 1.00 | (Reference) |  | 0.00 | (Reference) |
| <1 year | 1.57 | (0.58 to 4.20) |  | -0.07 | (-0.60 to 0.46) |
| 1 to 5 years | 0.82 | (0.37 to 1.83) |  | 0.24 | (-0.22 to 0.70) |
| >5 years | 0.89 | (0.32 to 2.46) |  | -0.27 | (-1.01 to 0.47) |
| Visits to doctor in last 12 months, quintiles | 0.82 | (0.65 to 1.03) |  | -0.13 | (-0.25 to 0.00) |
| ^a^Other tobacco use includes current regular use of E-cigarettes, cigars, pipes, snuff, chewing tobacco, and/ or hookah. | | | | | |

**Supplemental Table 4.** Fully adjusted^a^ logistic regression models including odds ratios (OR) and 95% confidence intervals (CIs) with outcomes of total serum prostate specific antigen (PSA) defined by three separate cutpoints (2.5 ng/ mL, 4.0 ng/ mL and 10.0 ng/ mL) stratified by age (y); Chicago 2013-2018.

| **Participant Characteristics** | **Model with outcome:**  **PSA** ≥**2.5 ng/ mL** | |  | **Model with outcome:**  **PSA** ≥**4.0 ng/ mL** | | |  | **Model with outcome:**  **PSA** ≥**10.0 ng/ mL** | |
| --- | --- | --- | --- | --- | --- | --- | --- | --- | --- |
|  | **OR** | **(95% CI)** |  | **OR** | **(95% CI)** | |  | **OR** | **(95% CI)** |
| Age | 40 to <55 years | | | | | | | | |
| Cigarette smoking history | | |  |  | |  |  |  |  |
| Never | 1.00 | (Reference) |  | 1.00 | (Reference) | |  | ~ | ~ |
| 0 to 1 pack-year | 1.29 | (0.50 to 3.37) |  | 1.16 | (0.29 to 4.72) ^*^ | |  | ~ | ~ |
| >1 pack-year | 1.48 | (0.37 to 6.49) |  | 1.76 | (0.18 to 16.8)^**^ | |  | ~ | ~ |
| Current marijuana use | | | | |  | |  |  |  |
| No | 1.00 | (Reference) |  | 1.00 | (Reference) | |  | ~ | ~ |
| Yes | 1.43 | (0.63 to 3.23) |  | 1.67 | (0.47 to 5.92) | |  | ~ | ~ |
| Other current tobacco use^b^ | | | | |  | |  |  |  |
| No | 1.00 | (Reference) |  | 1.00 | (Reference) | |  | ~ | ~ |
| Yes | 1.05 | (0.35 to 3.11) |  | 2.32 | (0.56 to 9.59) | |  | ~ | ~ |
| Total | 497 | | | | | | | | |
| Age | ≥55 years | | | | | | | | |
| Cigarette smoking history | | | | |  | |  |  |  |
| Never | 1.00 | (Reference) |  | 1.00 | (Reference) | |  | 1.00 | (Reference) ^*^ |
| 0 to 1 pack-year | 1.52 | (0.76 to 3.04) |  | 2.26 | (0.76 to 6.73)^**^ | |  | 4.25 | (0.35 to 51.4) ^*^ |
| >1 pack-year | 1.49 | (0.66 to 3.33) |  | 5.09 | (1.57 to 16.6)^**^ | |  | 3.68 | (0.20 to 66.9) ^*^ |
| Current marijuana use | | | | |  | |  |  |  |
| No | 1.00 | (Reference) |  | 1.00 | (Reference) | |  | 1.00 | (Reference) ^*^ |
| Yes | 0.49 | (0.22 to 1.09) |  | 0.27 | (0.08 to 0.96)^**^ | |  | 0.33 | (0.03 to 4.36) ^*^ |
| Other current tobacco use^b^ | | | | | | |  |  |  |
| No | 1.00 | (Reference) |  | 1.00 | (Reference) | |  | 1.00 | (Reference) |
| Yes | 1.02 | (0.39 to 2.68) |  | 2.38 | (0.76 to 7.52) ^*^ | |  | 13.1 | (2.09 to 82.3)^**^ |
| Total | 431 | | | | | | | | |
| Age | All ages | | | | | | | | |
| Cigarette smoking history | | | | | | |  |  |  |
| Never | 1.00 | (Reference) ^**^ |  | 1.00 | (Reference)^**^ | |  | 1.00 | (Reference) |
| 0 to 1 pack-year | 1.48 | (0.87 to 2.52) |  | 1.86 | (0.84 to 4.12)^**^ | |  | 2.15 | (0.34 to 13.5) ^*^ |
| >1 pack-year | 1.74 | (0.89 to 3.41) |  | 4.34 | (1.73 to 10.9)^**^ | |  | 2.45 | (0.22 to 27.5) ^*^ |
| Current marijuana use | | | | | | |  |  |  |
| No | 1.00 | (Reference) ^**^ |  | 1.00 | (Reference) ^*^ | |  | 1.00 | (Reference) ^*^ |
| Yes | 0.76 | (0.44 to 1.30) |  | 0.55 | (0.25 to 1.22) ^*^ | |  | 0.36 | (0.04 to 3.19) ^*^ |
| Other current tobacco use^b^ | | | | | | |  |  |  |
| No | 1.00 | (Reference) ^**^ |  | 1.00 | (Reference) ^*^ | |  | 1.00 | (Reference) |
| Yes | 0.94 | (0.47 to 1.88) |  | 1.99 | (0.91 to 4.38) ^*^ | |  | 6.62 | (1.56 to 28.1)^*^ |
| Total | 928 | | | | | | | | |
| ^a^ Adjusted for age (continuous), marital status, individual and neighborhood socioeconomic status, self-reported health, previous cancer diagnosis, body mass index, hypertension medication (yes/ no), health insurance type, timing of last prostate specific antigen test, timing of last prostate exam such as digital rectal exam, and visits to a doctor in the last 12 months (quintiles).  ^b^ Other tobacco use includes current regular use of E-cigarettes, cigars, pipes, snuff, chewing tobacco, and hookah. | | | | | | | | | |
| ^*^p-value < 0.05  ^**^p-value <0.01  ~ Statistic suppressed due to outcome frequency <10 | | | | | | |  |  |  |
